# Telemedicine versus conventional care for nonspecific neck pain: a randomized controlled study

**DOI:** 10.55730/1300-0144.6094

**Published:** 2025-09-25

**Authors:** Selkin YILMAZ MULUK, Sevtap BADIL GÜLOĞLU, Begüm ÜNLÜTAŞKIRAN

**Affiliations:** 1 Department of Physical Medicine and Rehabilitation, Ministry of Health Antalya City Hospital, Antalya, Turkiye; 2 Department of Physical Medicine and Rehabilitation, Antalya Training and Research Hospital, Antalya, Turkiye

**Keywords:** Telemedicine, neck pain, physical and rehabilitation medicine, exercise therapy, pain measurement

## Abstract

**Background/aim:**

Telemedicine (TM) can overcome travel barriers in nonspecific neck pain (NNP), yet a comprehensive, physiatrist-supervised TM model has not been directly compared to standard in-clinic care. The aim of this study was to determine whether a video-based exercise and remote follow-up program is as effective as conventional follow-up in reducing pain and disability in NNP.

**Materials and methods:**

This parallel-group randomized controlled trial (registered in ClinicalTrials.gov) included 68 adults (mean age = 40.52 years) with NNP who were randomly allocated 1:1 to the TM group (TG, n = 34) or control group (CG, n = 34). Both received identical exercises via prerecorded video (TG) or printed brochure (CG) to be performed for 3 months. TG visits were via video call; CG visits were in the clinic. Primary outcomes were pain measured by the visual analog scale (VAS) at baseline, day 15, month 1, and 3, and disability measured by the neck disability index (NDI) at baseline and month 3. Secondary outcomes were exercise adherence, patient satisfaction, and travel/time burden. Analyses followed the intention-to-treat principle using linear mixed models.

**Results:**

Both groups achieved clinically important VAS reductions (TG: 6.56 to 3.02, CG: 6.44 to 2.96). The only between-group difference appeared at day 15, favoring CG (p = 0.038) and the group × time interaction was nonsignificant overall (p = 0.111). NDI improved similarly in both groups (p = 0.473). Adherence in TG and CG (67.90% and 71.21%, respectively) and satisfaction (4.25 and 4.42, respectively) were comparable. TG participants avoided a mean of 38.88 km/175.74 min of travel across 3 virtual visits.

**Conclusion:**

A structured TM program with remote physiatrist follow-up matches conventional outpatient care in managing NNP. Larger, long-term studies should define optimal support and assess cost effectiveness across diverse populations.

## 1. Introduction

Neck pain is a prevalent global health concern and a leading cause of disability. It is most commonly classified as nonspecific neck pain (NNP) when no identifiable pathology, such as infection, tumor, fracture, or radiculopathy, is present [1,2]. NNP is characterized by discomfort or pain located between the upper nuchal line and the spinous process of the first thoracic vertebra, and may radiate to the head, shoulders, or upper limbs [1]. Its lifetime prevalence ranges from 22% to 86.8%, with 1-year incidence rates between 10.4% and 21.3%. At any given time, 10–20% of individuals report neck symptoms. The condition is more common in women around the fifth decade in high-income and urban populations [1,3]. Globally, 203 million people were affected by neck pain in 2020, and projections indicate a 32.5% increase in cases by 2050 [4].

Exercise therapy (particularly coordination, strengthening, and endurance exercises), patient education, and manual techniques are helpful in NNP management [5,6]. Home-based exercise programs are widely used for their accessibility and effectiveness, typically taught in person and reinforced with brochures. However, instruction retention and long-term adherence remain suboptimal, and repeated clinic visits add cost and time burdens.

Given these limitations, telemedicine (TM) offers a promising solution. It allows healthcare providers to deliver video-based exercise programs with remote follow-up. This approach enables patients to rewatch instructions, reinforce learning, reduce travel-related barriers, and may lead to improved clinical outcomes.

Recent reviews show telerehabilitation effectively reduces pain and improves function in NNP [7,8]. Although most studies have evaluated telerehabilitation or teleexercize alone, none have compared a comprehensive, physiatrist-supervised TM program—with asynchronous exercise delivery and scheduled remote follow-ups—against standard care [9–11]. Previous studies have tested isolated telerehabilitation components (e.g., phone calls, smartphone apps, or videoconferencing) without structured physician follow-up [7]. These approaches do not reflect real-world outpatient practice, where exercises are prescribed and reinforced through regular physician consultations. In contrast, our study evaluated a model that combines asynchronous video-based exercise delivery with scheduled, physiatrist-led teleconsultations, thus mimicking the traditional clinic setting in person via a TM platform.

To address this gap, we designed a randomized controlled trial comparing a comprehensive, physiatrist-supervised TM program with conventional outpatient care in adults with NNP. We hypothesized that both groups would improve, but the TM approach might provide additional benefits in pain reduction, functional outcomes, exercise adherence, and patient satisfaction by enhancing accessibility and reducing travel-related burden.

## 2. Materials and methods

### 2.1. Study design

The study was retrospectively registered in ClinicalTrials.gov as NCT06818422 and approved by the Ethical Committee of Antalya Education and Research Hospital (number: 11/9, date: 8 August 2024). It was a 2-center, parallel-group randomized clinical study that used 1:1 individual randomization. It compared TM with standard follow-up in adults with ≥3-month NNP. Informed consent was obtained from all participants.

No modifications were made to the study protocol, sample size, outcome measures, or data collection methods after the trial began. The protocol set a target sample of 80 and scheduled evaluations at baseline, day 15, and months 1 and 3. Recruitment occurred in outpatient physiatry clinics at 2 tertiary care centers. Although there was no formal patient or public involvement in the trial design, conduct, or reporting, the intervention content and follow-up procedures were informed by clinical experience and feedback from routine patient care.

**Figure 1:**
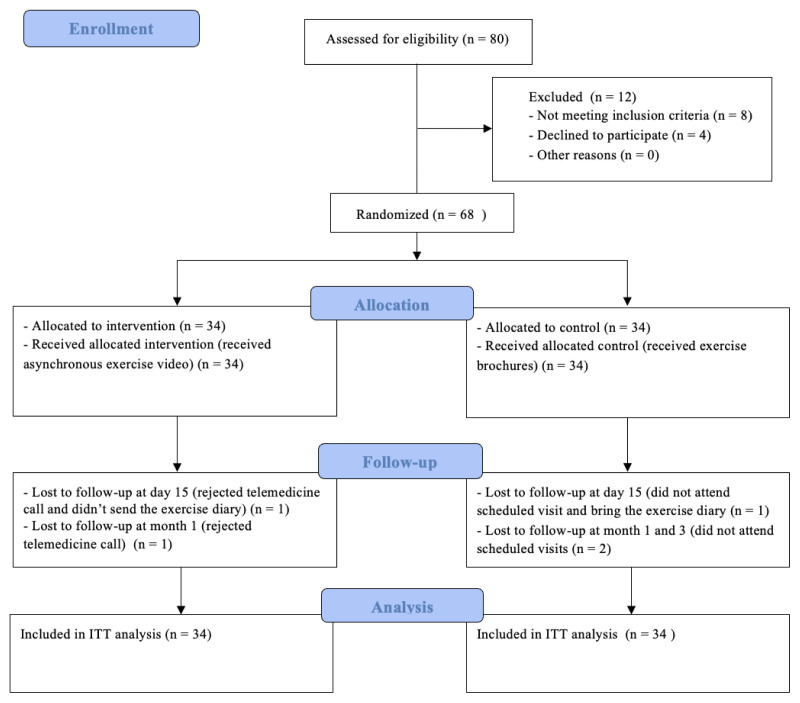
CONSORT flow diagram of the study.

Of 80 individuals screened, 12 were excluded (8 were ineligible and 4 declined), and 68 were randomized equally to intervention and control groups. All 34 in the intervention group received an asynchronous exercise video; all 34 in the control group received printed brochures (Figure 1). Missed follow-up assessments were retained in the intention-to-treat (ITT) analysis. The trial was reported per the CONSORT 2025 expanded checklist [12].

### 2.2. Eligibility criteria for participants

Adults aged 18 to 65 years who presented to outpatient physiatry clinics and with ≥3 months of NNP (e.g., cervicalgia, cervical straightening, arthrosis, or discopathy) without neurological deficits were included if they had the cognitive and physical ability to perform home exercises, basic digital literacy, and access to a smartphone or tablet with a stable internet connection. Exclusion criteria included cognitive or severe psychiatric disorders, red-flag symptoms (e.g., recent trauma, malignancy, or cervical canal stenosis), prior cervical surgery or injection within the past 3 months, recent physical therapy for neck pain, lack of digital access, visual impairment, or serious comorbidities such as advanced cancer. Participants were recruited from outpatient physiatry clinics via routine referrals by physicians.

### 2.3. Eligibility criteria for sites and personnel

The trial ran in 2 tertiary hospitals with physical medicine and rehabilitation departments capable of outpatient recruitment and digital follow-up. A prerecorded, standardized exercise video and brochure eliminated the need for extra staff training. A physiatrist performed all outcome assessments and data entry, and every patient contact—remote or in person—was handled by experienced physiatrists using a uniform protocol to maintain consistency.

### 2.4. Intervention and comparator

The TM group (TG) received a prerecorded video containing exercise demonstrations and NNP education, covering coordination, strengthening, and endurance exercises; the video could be rewatched as needed. The control group (CG) received the same content in a printed brochure with illustrations and text but without the audiovisual or replay features. Participants were instructed to perform the exercises 3 times daily for 3 months.

During the first 2 weeks, all participants were instructed to track their exercises using a structured diary. TG participants submitted diary photos via WhatsApp on day 15, while CG participants brought their diaries to the clinic visit that day. Follow-ups were conducted via video calls for the TG and in person for the CG at day 15, month 1, and month 3.

No tailoring of the intervention was applied. No cointerventions were permitted during the study period. Materials used in the intervention are available upon request from the corresponding author.

### 2.5. Outcome measures

Primary outcomes were pain, measured by the visual analog scale (VAS) (0–10 cm), and neck function, measured by the neck disability index (NDI) (0–50), analyzed as changes from baseline. VAS was assessed at baseline, day 15, month 1, and month 3; NDI at baseline and month 3. Both the VAS and NDI are validated and reliable outcome measures, including validated local language adaptations of the NDI [13–16]. Due to differing follow-up formats (remote vs in person), assessors could not be fully blinded, though standardized procedures were consistently applied. Secondary endpoints included exercise adherence on day 15 (completed/expected diary sessions), patient satisfaction at month 3 (5-point Likert scale: 0 = not satisfied, 5 = very satisfied), and baseline travel burden, measured by self-reported distance (km) and time (min) to the hospital.

All outcomes were predefined, and their selection was based on clinical relevance to evaluating treatment adherence, feasibility, and patient-centered outcomes. Both VAS and NDI are well established and recommended as core outcome measures in musculoskeletal pain trials.

Given the noninvasive nature of the intervention, no specific adverse events were anticipated. No harms were reported, and no participants discontinued the intervention due to adverse outcomes during the trial.

### 2.6. Data collection

Baseline evaluations were performed in person for all participants. Follow-up assessments were conducted via video calls for the TG and through in-clinic visits for the CG. Exercise adherence was documented using structured diaries, submitted digitally (TG) or in print (CG). Patient satisfaction, travel distance, and time burden were collected via self-reported questionnaires.

### 2.7. Sample size

The sample size calculation was based on the primary outcome (VAS score) using the minimum clinically important difference (MCID) of 18 mm (95% CI: 16–20) reported by Todd and Funk [17]. While this suggested a large effect size (d = 1.56) with a pooled SD of 11.5, a more conservative estimate of d = 0.7 was used to avoid overestimation. An a priori power analysis (G*Power version 3.1; 2-tailed t-test, α = 0.05, power = 0.80, 1:1 allocation) indicated a required sample of 34 per group (n = 68). To allow for potential dropouts, the recruitment target was increased. Of 80 patients screened, 12 were excluded (8 were ineligible and 4 declined), and 68 were randomized equally into the TG and CG.

No interim analyses were planned or conducted, and no stopping guidelines were established, given the minimal risk associated with the noninvasive intervention. All participants were retained for follow-up as per the original protocol.

### 2.8 Randomization and blinding

Eligible participants were randomly assigned in a 1:1 ratio to either the TG or the CG. An age- and sex-stratified block-randomization list was generated with Random.org. An unrelated staff member kept the list and revealed group assignments after baseline assessment; sealed envelopes ensured allocation concealment. Then, participants were enrolled.

Participants were partially blinded: they knew their own follow-up format but were not told that an alternative group existed or what it received. Outcome assessors were blind only at baseline; later assessments (remote vs in person) precluded assessor blinding, although VAS and NDI were self-reported to minimize observer bias. Data analysts/statisticians worked with anonymized group codes. The randomization list was inaccessible throughout the study.

Due to differences in delivery (video/remote vs brochure/in person), an identical intervention was not possible. However, content was fully standardized, and both groups received equal contact frequency and duration. No emergency unblinding occurred during the trial, and there were no known compromises to the blinding procedures.

### 2.9. Statistical analysis

Analyses followed the ITT principle and were run using SPSS (version 30.0, IBM Corp., Armonk, NY, USA) and Jamovi (version 2.6, Sydney, Australia; https://www.jamovi.org). Shapiro–Wilk tests informed the use of independent t-tests for normally distributed data and Mann–Whitney U for nonnormally distributed or ordinal variables. Pain (VAS: baseline, day 15, month 1, and month 3) and neck function (NDI: baseline and month 3) were modeled with linear mixed effects (random intercept; fixed group, time, group × time). Prior to conducting linear mixed-effects modeling, key assumptions were evaluated. Normality of residuals was assessed via Q–Q plots and the Shapiro–Wilk test. Homoscedasticity and linearity were examined through residual vs fitted plots. All assumptions were met, confirming the validity of the model estimates. Satterthwaite df, 2-tailed α = 0.05, and reporting of Cohen’s d with 95 % CIs were used; Bonferroni-adjusted pairwise tests explored significant time effects. Because 4 time points were analyzed for VAS and 2 for NDI, the total number of within-group pairwise comparisons did not exceed six. Thus, the Bonferroni correction was considered appropriate and sufficiently conservative to control for Type I error.

Missing data were addressed within the mixed models under a missing at random (MAR) assumption (no imputation). Multiplicity control was limited to Bonferroni; no additional subgroup or sensitivity analyses were planned beyond the age/sex stratification. The primary outcome analysis was prespecified, and posthoc within-group/time contrasts are presented with adjusted p-values. Exercise adherence was calculated as completed sessions / recommended sessions) × 100.

## 3. Results

A total of 68 participants were randomized equally to the TG and CG, all receiving their assigned interventions and completing follow-ups. No exclusions occurred. Groups were comparable in baseline characteristics, with no significant differences in age, baseline VAS, baseline NDI, pain duration, or BMI (all p > 0.05). The sample was predominantly female, university-educated, and married. Additional demographics are detailed in Table 1. Because baseline characteristics (age, sex, baseline VAS/NDI, and BMI) were statistically comparable across groups with small or negligible effect sizes (Table 1), no covariates were included in the primary model to avoid unnecessary overadjustment.

All analyses followed the ITT principle, with missing data accommodated by a linear mixed model (LMM) (random intercept; fixed group, time, and group × time) under an MAR assumption. Model fit was acceptable (conditional R^2^ = 0.601; marginal R^2^ = 0.266). The overall group × time effect was not significant (F = 2.03, p = 0.111), but a significant between-group difference emerged at day 15, favoring the CG (p = 0.038). Full estimates are shown in Table 2.

**Figure 2:**
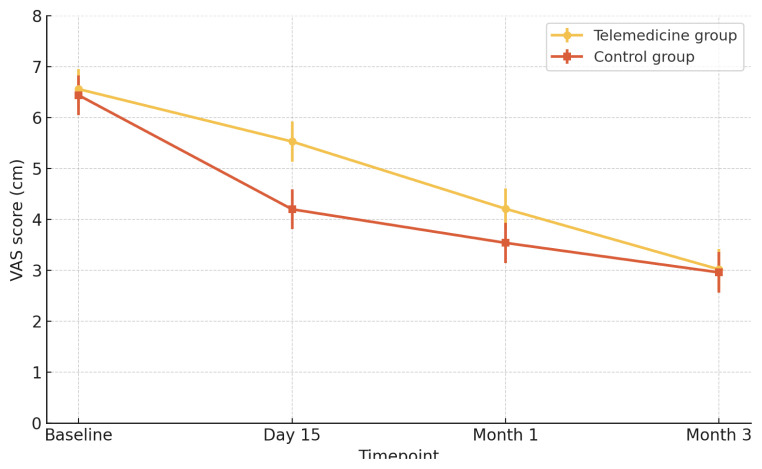
Change in VAS scores over time by group.

Estimated marginal means (EMMs) (Table 3) and the trajectory plot (Figure 2) show a clear time effect: VAS scores fell steadily in both arms from baseline to month 3. The sole between-group difference appeared on day 15, where the CG reported lower pain than the TG (p = 0.038). At all other time point assessments, the 95% CIs overlapped and p-values exceeded 0.05, indicating that the early advantage was not sustained. Posthoc contrasts confirmed substantial within-group gains—with the largest effect from baseline to month 3 (Cohen’s d = 1.45)—and moderate-to-large improvements for the other time intervals; full statistics are provided in Supplementary Table 1. Effect sizes were calculated to quantify the magnitude of within- and between-group differences. Cohen’s d was used for normally distributed continuous variables, interpreted as small (0.2), medium (0.5), or large (0.8). For nonparametric tests, effect size r was reported (small = 0.1, medium = 0.3, or large = 0.5). All reported effect sizes are presented alongside 95% CIs in Supplementary Table 1. Overall, both groups experienced clinically meaningful pain reduction, with no lasting superiority of either follow-up method.

Regarding the neck disability, LMM analysis showed a significant main effect of time (p < 0.001), indicating substantial improvement across both groups. No significant group effect or group × time interaction was observed (p = 0.973 and p = 0.473, respectively), as summarized in Table 4.

**Figure 3:**
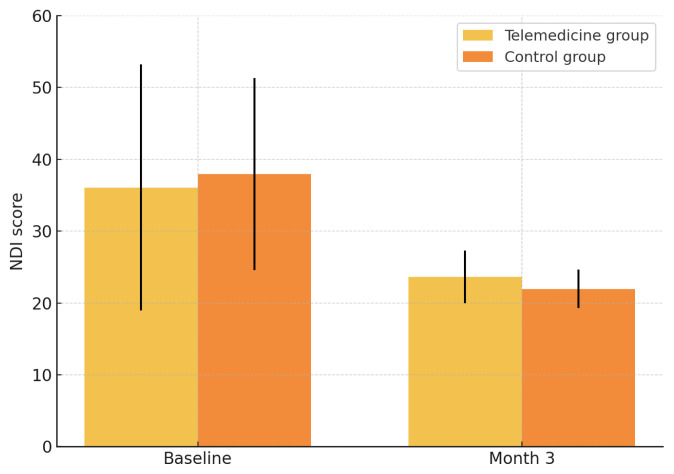
EMM of NDI scores at baseline and month 3 by group.

EMMs for NDI are shown in Table 5 and Figure 3. Disability fell in both groups from baseline to month 3, yet between-group differences remained nonsignificant at both assessments (p = 0.302 and 0.473 at baseline and month 3, respectively) with overlapping 95% CIs, indicating comparable improvement. NDI was measured only at baseline and month 3, so no posthoc contrasts were needed.

Exercise adherence, reported by 66 participants, was 67.90% (range: 30.95–100.00) in the TG and 71.21% (range: 28.57–100.00) in the CG. The difference was not statistically significant (U = 529.5, Z = −0.195, p = 0.846). Patient satisfaction was reported by 63 participants. Mean scores were 4.25 (SD = 0.80) in the TG (n = 32) and 4.42 (SD = 1.03) in the CG (n = 31), with no significant between-group difference (U = 407.0, Z = −1.371, p = 0.170).

Travel and time burden were evaluated in the TG, where participants lived or worked a mean of 6.48 km (SD = 4.51) from the hospital, requiring an average of 29.29 min (SD = 23.12) per visit. The TM approach saved an estimated 38.88 km and 175.74 min of travel per participant across 3 virtual follow-ups.

During both TM and in-person visits, patients most frequently asked about exercises, posture, and ergonomics, all of which were addressed by the physiatrist. No adverse events or unintended effects occurred in either group during the study.

## 4. Discussion

### 4.1. Interpretation of findings

This trial compared a TM follow-up program with conventional in-clinic care for NNP. As hypothesized, both groups achieved significant, clinically meaningful reductions in pain and disability over 3 months; VAS score decreases were greater than MCID. However, TM was not superior as expected. The only between-group difference was lower VAS in the control group at day 15 (p = 0.038), but that disappeared by months 1 and 3, indicating a transient finding. The TM group was also no better in exercise adherence, patient satisfaction, or final NDI scores. Similar outcomes probably reflect the identical exercise content and clear, structured guidance given to both groups, producing high adherence across treatment programs.

The transient advantage of the control group at day 15 may have several explanations. Some patients might have perceived brochures as more straightforward during the initial weeks, or occasional internet interruptions or minor technical barriers could have limited optimal video use early on. However, given that asynchronous video was simple and readily available on patients’ smartphones or tablets, the difference is unlikely to reflect a true practical advantage of brochures. Considering that the effect disappeared by months 1 and 3 and the overall group × time interaction was not significant, a chance finding remains the most plausible explanation.

Overall, home-based exercise plus structured physician follow-up improved pain and function equally well in both study groups, showing that exercise combined with structured physician follow-up improved pain and function, regardless of whether care was delivered by TM or in person.

### 4.2. Benefits and harms

From a clinical implementation perspective, the main driver of improvement in both groups was the structured exercise program, which led to substantial reductions in pain and disability. TM did not add further clinical benefit beyond exercise, but it offered a clear logistical advantage: participants in the intervention group avoided considerable travel distance and time through remote follow-up visits. This benefit is particularly relevant for individuals with mobility limitations, transportation challenges, or limited access to care. No adverse events or harms were reported in either group, supporting the safety of the exercise-based approach regardless of delivery mode.

### 4.3. Comparison with existing evidence

Our results reflect some prior trials of telerehabilitation for NNP. A study that delivered spinal-stabilization exercises either remotely or face-to-face reported equivalent improvements in pain, disability, muscle architecture, and body awareness, mirroring the equal efficacy we observed between formats [18]. Similarly, a trial that compared telerehabilitation with manual therapy and identical home exercises with manual therapy also found no outcome differences, underscoring the feasibility of remote care for musculoskeletal disorders [10]. A third trial reached mixed conclusions: pain relief (VAS) was again comparable between formats, yet the telerehabilitation arm achieved larger improvements in disability (NDI) and higher exercise adherence [19].

Some trials have reported clearer advantages for telerehabilitation. For example, Barbosa et al. [11] showed that adding video-guided exercise to an online booklet produced significantly greater reductions in pain and disability than the booklet alone. Likewise, a 6-month study in chronic neck pain found home-based TM more effective than standard care in lowering both pain and disability, suggesting remote delivery may offer added long-term benefit [20].

Unlike earlier trials that concentrated mainly on remote exercise delivery, our study assessed a broader, physiatrist-supervised TM model that relied chiefly on scheduled physician–patient follow-ups, thereby recreating the clinical oversight typical of standard outpatient care.

Evidence from other specialties also supports the safety and efficiency of TM. In cardiology, remote physician-led follow-up for pacemaker patients reduced clinic visits without increasing adverse events [21]. Postprostatectomy video consultations provided outcomes comparable to in-person care while cutting travel time and costs [22]. Similarly, brief video visits after thyroid or parathyroid surgery allowed safe medication adjustments and laboratory monitoring, with high patient satisfaction [23]. In rheumatology, phone and web-based follow-ups lowered visit frequency and costs, although barriers remain for patients with hearing, cognitive, or technological limitations [24].

TM is expected to be increasingly used by outpatient, nonsurgical clinicians with high technical confidence. While it reduces patient barriers and offers convenience, its main drawback remains technical difficulties [25]. Siddiqui et al. [26] identified key barriers to TM in physiatry as difficulty in performing physical examinations, limited patient access to technology, and low digital literacy. They recommend improving provider training, digital literacy, and using secure hospital platforms to support future use.

Overall, TM provides results similar to, and sometimes better than, conventional care. It can support low-risk musculoskeletal conditions, helping reduce time and travel demands. However, issues like technical problems, low digital literacy, and the difficulty of doing physical examinations remotely still need to be addressed.

### 4.4. Limitations

Firstly, the sample size, while adequately powered for detecting differences in VAS scores, may have limited our ability to detect smaller effects. Secondly, although adherence diaries were used, they relied on self reporting, which may introduce recall bias or overestimation. Thirdly, the intervention formats were inherently different in presentation and may have introduced perception bias. Furthermore, generalizability is limited: participants were predominantly female and mostly university graduates, reflecting a subgroup that is more digitally literate than the general population. This may restrict the applicability of our findings to men, individuals with lower educational attainment, and those with limited internet access. Finally, although the 3-month follow-up was sufficient to capture short-term treatment effects, it does not allow conclusions about the long-term sustainability of telemedicine interventions for neck pain.

## 5. Conclusion

In this randomized controlled trial, adults with NNP improved substantially in pain and disability following a structured exercise program with regular physician follow-up. Clinical outcomes were comparable whether the program was delivered through TM or conventional in-person visits, indicating that TM can serve as an effective delivery method rather than a distinct treatment. Both groups showed high adherence, strong satisfaction, and no adverse events, while the TM group additionally benefited from reduced travel and time burden.

Future research should identify the best exercise formats, support levels, and program structures, extend follow-up beyond 6–12 months, include diverse age groups and regions, and compare cost effectiveness. If further studies confirm these benefits, TM may evolve from an alternative approach into a standard component of musculoskeletal pain management.

## Supplementary Information

**Supplementary Table 1:** Posthoc comparisons of VAS scores with effect sizes.

Comparison	Mean difference	SE	Cohen’s d	95% CI lower	95% CI upper
FirstVAS vs. SecondVAS	1.635	289	0.686	1.069	2.201
FirstVAS vs. ThirdVAS	2.623	292	1.089	2.051	3.195
FirstVAS vs. FourthVAS	3.506	294	1.446	2.930	4.082
SecondVAS vs. ThirdVAS	988	293	0.409	0.414	1.562
SecondVAS vs. FourthVAS	1.871	295	0.769	1.293	2.449
ThirdVAS vs. FourthVAS	883	296	0.362	0.303	1.463

VAS: Visual Analog Scale. Effect sizes calculated using Cohen’s d.

**Supplementary Table 2:** Normality tests for the demographic variables, baseline VAS, and NDI of the telemedicine and control groups.

	Statistic TG / CG	df	Sig. TG / CG
Age	0.947/0.946	34	0.103/0.092
BMI	0.965/0.939	34	0.335/0.059
Baseline VAS	0.951/0.964	34	0.130/0.308
Baseline NDI	0.915/0.936	34	0.012/0.046
Duration of pain (mo)	0.866/0.763	34	<0.001/<0.001

Normality was assessed using the Shapiro–Wilk test. TG: Telemedicine group; CG: control group; VAS: visual analog scale; NDI: neck disability index.

**Supplementary Table 3:** Baseline comparison of continuous variables between telemedicine and control groups.

Variable	Mean age TG	Mean age CG	p value	95% CI	Effect size
Age	40.32	40.71	0.903	−6.603 to 5.839	−0.030
Baseline VAS	6.56	6.44	0.799	−0.802 to 1.037	0.062
Baseline NDI	36.08	37.95	0.302	−0.117, 0.353	0.125
Duration of pain	20.42	23.38	0.820	−0.212, 0.264	0.028

For comparisons of age and baseline VAS variables independent samples t-test, for baseline NDI and pain duration variables Mann–Whitney U test is utilized according to Shapiro–Wilk results.

## Figures and Tables

**Table 1:** Descriptive statistics.

Variable	Telemedicine group (n:34) Mean ± SD (min-max)	Control group (n:34) Mean ± SD (min-max)	p value

Age	40.32 ± 13.39 (18–64)	40.71 ± 12.28 (20–64)	0.903

Body mass index	27.78 ± 4.97	26.50 ± 5.28	0.307

Baseline VAS	6.56 ± 2.22 (2–10)	6.44 ± 1.50 (3–10)	0.799

Baseline NDI	36.08 ± 17.12 (14.00–77.78)	37.95 ± 13.39 (18.00–70.00)	0.302

Duration of pain	20.42 ± 18.74	23.38 ± 26.89	0.820

Sex	28 (82.4%) female	27 (79.4%) female	
	6 (17.6%) male	7 (20.6%) male	

Education Level	3 (8.8%) ps	3 (8.8%) ps	
	12 (35.3%) hs	8 (23.5%) hs	
	19 (55.9%) univ	23 (67.6%) univ	

Marital status	25 (73.5%) married	25 (73.5%) married	
	9 single (26.5%)	9 single (26.5%)	

Heavy lifting	11 (32.4 %)	17 (50 %)	

Desk job	12 (35.3 %)	16 (47.1%)	

Travel job	3 (8.8 %)	1 (2.9 %)	

VAS: visual analog scale, NDI: neck disability index, ps: primary school, ss: secondary school, hs: high school, univ: university. Normality assessed via Shapiro–Wilk (n = 34/group); age and baseline VAS analyzed with independent samples t-test, NDI and pain duration with Mann–Whitney U tests (Supplementary Tables 2, 3).

**Table 2:** Fixed effects summary for VAS scores.

Predictor	Estimate (β)	SE	95% CI	t-value	p-value	Effect size (Cohen’s d)
Intercept	4.559	0.213	4.14 to 4.978	21.401	<0.001	2.730
Group (CG vs. TG)	−0.543	0.426	−1.38 to 0.296	−1.276	0.207	−0.325
Day 15 vs. baseline	−1.635	0.289	−2.20 to −1.066	−5.656	<0.001	−0.979
Month 1 vs. baseline	−2.623	0.292	−3.20 to −2.048	−8.983	<0.001	−1.571
Month 3 vs. baseline	−3.507	0.294	−4.08 to −2.928	−11.946	<0.001	−2.100
Group × Day 15	−1.206	0.578	−2.34 to −0.067	−2.085	0.038	−0.722
Group × Month 1	−0.553	0.584	−1.70 to 0.597	−0.947	0.345	−0.331
Group × Month 3	0.556	0.587	−1.10 to 1.212	0.095	0.924	0.033

CI: confidence interval; CG: control group; TG: telemedicine group.

**Table 3:** Estimated marginal means for visual analog scale scores.

Time point	Telemedicine group (Mean ± SE)	Control group (Mean ± SE)	Mean Difference (95% CI)	p-value	Effect Size (Cohen’s d)
Baseline	6.56 ± 0.388	6.44 ± 0.388	0.12 (−0.76, 1.01)	0.799	0.054
Day 15	5.53 ± 0.392	4.20 ± 0.392	1.33 (0.32, 2.34)*	0.038*	0.722
Month 1	4.21 ± 0.397	3.54 ± 0.397	0.67 (−0.34, 1.68)	0.345	0.331
Month 3	3.02 ± 0.397	2.96 ± 0.401	0.06 (−0.94, 1.07)	0.924	0.033

CI: confidence interval.

* Statistically significant at p < 0.05.

**Table 4:** Fixed effects summary for NDI scores.

Predictor	Estimate (β)	SE	95% CI	t-value	p-value	Effect size (Cohen’s d)
Intercept	29.768	1.670	26.47 to 33.07	17.848	<0.001	2.146
Group (CG vs TG)	0.114	3.340	−6.49 to 6.72	0.034	0.973	0.008
Month 3 vs. baseline	−14.493	2.440	−19.32 to −9.66	−5.939	<0.001	−1.045
Group × Time	−3.521	4.880	−13.18 to 6.14	−0.721	0.473	−0.254

CI: confidence interval; CG: control group; TG: telemedicine group.

**Table 5:** Estimated marginal means for neck disability index.

Timepoint	Telemedicine group (Mean ± SE)	Control group (Mean ± SE)	Mean Difference (95% CI)	p-value	Effect size (Cohen’s d)
Baseline	36.08 ± 17.12 (SD)	37.95 ± 13.39 (SD)	−1.87 (−9.12, 5.38)	0.302	0.114
Month 3	23.64 ± 3.67 (SE)	21.96 ± 2.69 (SE)	−1.87 (−10.54, 6.80)	0.473	0.254

CI: confidence interval. NDI values at month 3 were not directly reported as marginal means; approximate values were inferred from the model summary. Baseline values are reported as mean ± standard deviation (SD). Month 3 values represent estimated marginal means ± standard error (SE), derived from the linear mixed-effects model.
